# FmrBT: A comprehensive data set of field metabolic rates, body mass and ambient temperature

**DOI:** 10.1038/s41597-025-05868-y

**Published:** 2025-09-29

**Authors:** Francisco de Castro

**Affiliations:** Agri-Food and BioSciences Institute. 18a Newforge Lane, Belfast, BT9 5PX UK

**Keywords:** Ecological modelling, Theoretical ecology

## Abstract

Understanding the energetics of animals in their natural environments is critical for ecological research, conservation, and modeling of population dynamics. I present a comprehensive dataset of field metabolic rates (FMR) of animals, body mass and ambient temperature, encompassing data from a wide range of species and ecosystems. The dataset is a compilation of FMR measurements reported in the scientific literature on more than 700 species, and offers a valuable resource for researchers in ecology, conservation biology, and evolution. By including body mass and temperature, it allows: 1) use it in large-scale analyses of allometric models of body mass, and 2) incorporate explicitly the effects of temperature, making it a useful tool for comparative studies, ecological modeling, and conservation assessments, particularly regarding the effects of climate change.

## Background & Summary

The metabolic rate of an animal is one of its fundamental physiological and ecological traits^1^. It is a measure of the energy consumed in all activities required for survival and reproduction^2^. Energy needs, in turn, determine food requirements and indirectly affect other processes such as predation rates^3,4^. In short, it reflects the ‘pace of life’ of a species^5^. Metabolic rates, as other biological rates, are strongly correlated with body mass, a relationship known as “Kleiber’s Law”^6^. This relationship raises the possibility to use body mass to predict population and community characteristics such as growth and mortality rates, population density, diversity, and others^1,7–9^.

Ambient temperature (hereafter ‘temperature’) is another factor to consider: higher temperatures accelerate all chemical reactions, including metabolic; thus, in general, higher metabolic rates can be expected at higher temperatures, which has special relevance in the context of climate change.

The FmrBT^10^ dataset is an extensive collection of Field Metabolic Rates (FMR) measurements from diverse animal taxa, including mammals, birds, reptiles, fish, invertebrates, and unicellular organisms. FMR is the rate at which an animal expends energy in its natural habitat, encompassing all its activities, such as locomotion and foraging, digestion, thermoregulation and, in some cases, reproduction^2^. Therefore, it represents a direct estimate of the total energy needs of the individual, and a more precise method than using a constant multiple of basal metabolic rate (BMR), which is a common practice^11^. Furthermore, by combining FMR with assimilation and production efficiencies (*e*_*a*_, *e*_*p*_) and calorie content of prey (*ε*), maximum feeding rate (*I*) can be estimated as: $I={FMR}/{e}_{a}\left(1-{e}_{p}\right)\varepsilon$ and, therefore, interaction strengths in predator-prey dynamic models (e.g. ^3,12^).

## Methods

I conducted a systematic literature search on-line, primarily from peer-reviewed scientific publications, through academic platforms: Google Scholar, Web of Science or Research Gate. Only studies that included the body mass of individuals and temperature were selected. The data set includes studies from the OxyRef database^13^ in which the activity of animals was classified as “routine”, the individuals were not starved and not exposed to any stress. Studies that involved forcing up the metabolic rates of individuals by e.g. subjecting them to a constant water current to measure maximum metabolism, were excluded because they do not represent normal field metabolic rate.

I used the Global Biodiversity Information Facility^14^ to retrieve taxonomic information from species names, which is usually the only taxonomic information reported in the articles.

Many studies included in the data set used the doubly labeled water method, which is particularly suitable for long periods of time, in individuals for which other types of calorimetric measurements would be difficult or impossible. Metabolic rates expressed as Oxygen consumption were transformed into kJoules using conversion factor of 1 ml O2 = 21.1 Joules^15^. To transform mass of oxygen to volume, if needed, I use a conversion factor of 0.75 ml/mg (assuming 20 °C and 1 atmosphere). Body mass is expressed as fresh weight. If only dry weight or carbon was specified in the source, I used a factor of 0.5 to transform from carbon to dry weight and a factor of 0.25 to transform the dry weight to fresh weight^16,17^.

When several individuals were included in the same experiment and not measured individually (e.g., a group of fish in a tank) the means of body mass and metabolic rate were included as a single data point. If several experiments were performed under different conditions (different temperatures, feed regimes, etc.), each experiment was considered as a separate data point. When a variable was expressed as a range, the midpoint was taken.

**Fig. 1:**
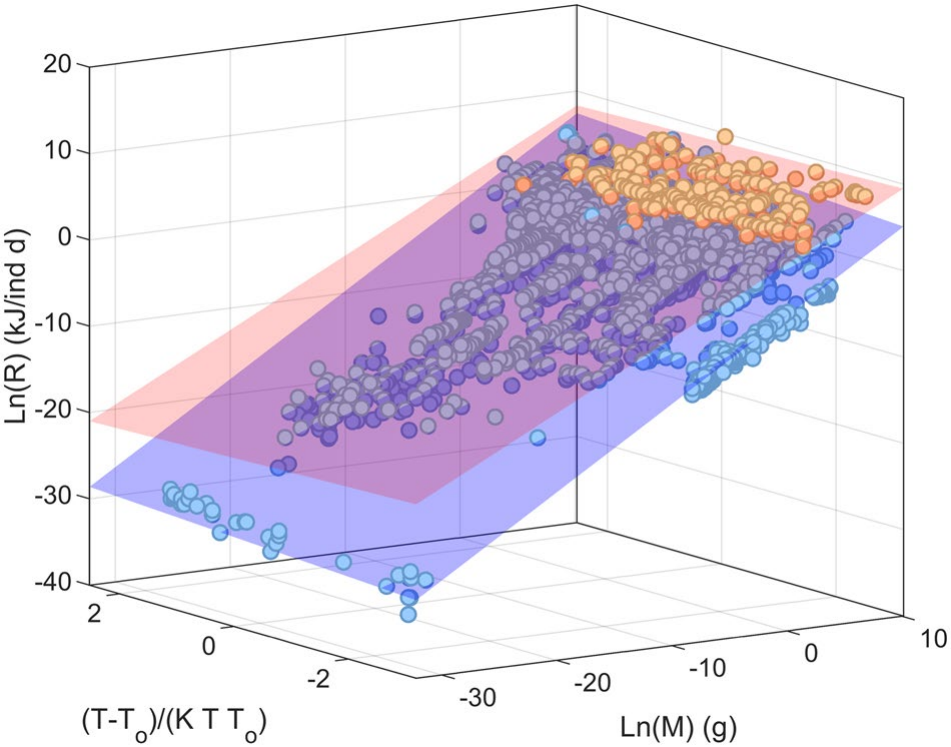
Fit of the Arrhenious model to data. *R* is field metabolic rate (kJ/ind d), *M* is body mass (g) and $\,(T-{T}_{o})/{KT}{T}_{0}$ is the Arrhenious temperature factor (see Table 1). The bluish colour is for Ectotherms and orange colour for endotherms.

To facilitate the application of the data, I fitted three models to them (Table 2, Fig. 1), all including body mass and three well known temperature factors^18^. The response variable is FMR (kJ/ind/d), and the predictors are body mass (grams) and ambient temperature (Celsius). The fitting was done separately for endotherms and ectotherms, in log-log scale using MATLAB function *fitlm*. All models reach the same R^2^, although there *was* a noticeable advantage in log-likelihood of the Arrhenious model for ectotherms. Notice that the temperature coefficient was positive for ectotherms but negative for endotherms, showing their differences in temperature regulation, which is important for modelling their response to climate change.

**Table 1:** Names and descriptions of variables in the dataset.

**Kingdom**	Kingdom (GBIF)
**Phylum**	Phylum (GBIF)
**Class**	Class (GBIF)
**Order**	Order (GBIF)
**Family**	Family (GBIF)
**Genus**	Genus (GBIF)
**SpeciesVerbatim**	Species name in source.
**SpeciesAcceptedName**	Acccepted species name (GBIF).
**FMR_kJ_d**	Field metabolic Rate in Kjoules per day per individual.
**Mass_g**	Individual body mass, in grams.
**Temp_C**	Study temperature as reported in source, in Celsius.
**Endotherm**	Boolean variable indicating if species is endothermic (1) or ectothermic (0).
**Reference**	Rerence of source: all authors or 1^st^ author, and year.
**Comment**	Comment in Thusrton and Gehrke
**Outlier**	Boolean variable indicating the record is an outlier (1) according to Cook’s distance

**Table 2:** Parameters and goodness of fit of the models.

		Arrhenious model	Belehradek model	Berthelot model
		${\boldsymbol{R}}{\boldsymbol{=}}{\boldsymbol{a}}{{\boldsymbol{M}}}^{{\boldsymbol{b}}}{{\boldsymbol{e}}}^{{\boldsymbol{E}}({\boldsymbol{T}}-{{\boldsymbol{T}}}_{{\boldsymbol{o}}})/{\boldsymbol{KT}}{{\boldsymbol{T}}}_{{\boldsymbol{0}}}}$	${\boldsymbol{R}}={\boldsymbol{a}}{{\boldsymbol{M}}}^{{\boldsymbol{b}}}{{\boldsymbol{T}}}^{{\boldsymbol{E}}}$	${\boldsymbol{R}}{\boldsymbol{=}}{\boldsymbol{a}}{{\boldsymbol{M}}}^{{\boldsymbol{b}}}{{\boldsymbol{E}}}^{{\boldsymbol{T}}}$
Ectoth.	Ln(a)	−2.11 [−2.15, −2.08]	−78.61 [−86.01, −71.13]	−15.56 [−16.89 −14.23]
	b	0.844 [0.839, 0.849]	0.844 [0.839, 0.849]	0.844 [0.839, 0.849]
	E	0.34 [0.31, 0.37]	13.47 [12.15, 14.78]	
	Ln(E)			0.046 [0.041, 0.050]
	Adj R^2^	0.966	0.966	0.966
	LogL	5998	3913	3913
Endoth.	Ln(a)	1.94 [1.80, 2.07]	48.94 [37.79, 60.09]	10.39 [8.42, 12.37]
	b	0.674 [0.650, 0.697]	0.674 [0.650, 0.698]	0.674 [0.651, 0.698]
	E	−0.204 [−0.25, −0.15]	−8.27 [−10.24, −6.31]	
	Ln(E)			−0.029 [−0.036, −0.022]
	Adj R^2^	0.938	0.938	0.938
	LogL	104.9	104.7	104.6

All models include a body mass factor and a temperature factor. *R*, the dependent variable, is metabolic rate (kJ/ind d), *M* is body mass (g), *T* is the ambient temperature (°K), and *K* is the Boltzmann constant (8.62 10^−5^ eV/K). a, b and E are the estimated parameters. In Arrhenious model, *T*_*0*_ is 293 °K (20 °C). The fitting was done in log-log using MATLAB function *fitlm*. Parameters *a* (in all models) and *E* (in Berthelot model) are shown as natural log. Confidence intervals (95%) shown between brackets. LogL is Log-likelihood. The number of data is 3914 for ectotherms and 234 for endotherms.

## Data Records

The dataset is at Zenodo^10^. At the time of publication, the dataset contained 4567 records from ~190 sources. In total it includes 719 identified species or equivalent (more unidentified) from 49 Classes. Contributions of new data or feedback to improve the dataset are welcome. The dataset is composed of three files:

1. An Excel file with the data on FMR, body mass and ambient temperature, and the taxonomy of each species (FieldMR_SciDataPub.xlsx). The columns of the table with names and units are specified in Table 1. This file contains a second sheet with a table with the DOI of most of the references obtained from CrossRef.org.
2. An EndNote file (deCastro_SciData_Refs.enl) with all the references from which the data were obtained.
3. A MATLAB script (deCastro_SciData_Fit.m) that performs the model fitting described above and produces Fig. 1.

## Technical Validation

The data were retrieved only from peer reviewed papers that reported original observations. Sources for which interpretation was doubtful, or from which raw measurements could not be extracted, were not included. Specific cases that did not fit the concept of field metabolic rate were not included (e.g. insects during flight). Unit conversions for body mass and metabolic rate were done manually for each source after data retrieval using the conversion factors described in methods. Further, after fitting the allometric models to the data (see methods), records with a Cook distance three times the mean^19^ (i.e. unusual after taking into account the effect of body mass and temperature) were removed from the fitting and checked against the original source for errors, such as wrong units or misplaced decimal point. These records were flagged in the data file. They represented ~ 7% of the data. The underlying manuscripts utilized for this work are refs. ^20–205^.

## Usage Notes

This dataset can be used to explore a wide range of biological and ecological questions, including:

1. Comparison of metabolic rates across taxa and ecosystems. This facilitates the identification of patterns in metabolic rates, helping to answer questions about the ecological and evolutionary factors that drive variation in energy expenditure.
2. It allows to estimate the energy requirements of individuals to understand energy flow in ecosystems. This information is important to improve bioenergetic models and better understanding resource allocation, competition, and predator-prey dynamics.
3. As the Earth’s climate changes, it is crucial to understand how animal metabolic rates may be affected. By explicitly including temperature as predictor, this dataset can serve to predict potential shifts in energy expenditure in response to changing conditions. This can be important in conservation and resource management. For species of economic importance, such as in fisheries or agriculture, understanding their metabolic rates can inform sustainable management practices and help in the long-term viability of these resources.

## Data Availability

The dataset is accessible online at Zenodo^10^ [10.5281/zenodo.16894769]. It consists of a single Excel file with the data, together with an EndNote file that contains all the references used in the data set, and a Matlab script with the model fitting. The variables and units in the data are detailed in Table 1.

## Code availability

The code used to fit the models, produce the figures and check for outliers is included in the Zenodo record^10^ (see below). It is a script in Matlab v. R2018b. To use it, open Matlab, navigate to the folder where the Excel data file and the script are and type the name of the script in the command window.
